# Exploring the burden of non-communicable diseases on surgical services in Africa: a comprehensive literature review

**DOI:** 10.1097/MS9.0000000000003236

**Published:** 2025-04-02

**Authors:** Poliana Zanotto Manoel, Olivier Uwishema, Agnes Zanotto Manoel, Innocent Chijioke Dike, Nour Yassin, Sarah Mshaymesh

**Affiliations:** a Department of Research and Education, Oli Health Magazine Organization, Kigali, Rwanda; b Department of Medicine, Faculty of Medicine, Federal University of Rio Grande, Rio Grande, Rio Grande do Sul, Brazil; c Department of Medicine, Federal Teaching Hospital Ido-Ekiti, Ekiti, Nigeria; d Department of Medicine, Faculty of Medicine, Beirut Arab University, Beirut, Lebanon; e Division of Natural Sciences, Faculty of Sciences, Haigazian University, Beirut, Lebanon

**Keywords:** Africa, education, health services, noncommunicable diseases, operative, surgical procedures

## Abstract

**Introduction:**

Non-communicable diseases (NCDs) encompass five major categories: cardiovascular disease, cancer, diabetes mellitus, mental health disorders, and chronic respiratory diseases. The burden of NCDs is rising in Africa, particularly in sub-Saharan Africa, having increased by 67% from 1990 to 2017, which puts additional pressure on healthcare services. Although most treatments for NCDs are non-surgical, surgical intervention plays a crucial role in managing many of these diseases. Unfortunately, barriers to accessing surgical care for NCDs remain in Africa.

**Methods:**

A comprehensive literature review was conducted employing the following search databases: AJOL, PubMed/Medline, and Google Scholar. Search strategies that combined terms pertaining to “non-communicable disease,” “surgical care,” and “Africa” were utilized.

**Results:**

The main barriers to surgical access in the management of NCDs in Africa include restricted financial resources, a lack of adequate infrastructure, and deficiencies in competent surgical training. Measures to improve surgical intervention associated with NCDs include international collaborations, improved education and training of professionals, and the use of mobile technology tools.

**Discussion:**

There is a need to implement strategies that seek to improve access to surgery for the treatment of NCDs on the African continent. Some of these strategies involve multi-professional action alongside the establishment of prevention actions and policies aimed at the management of NCDs. Furthermore, support from the governments of each nation, as well as ongoing research on the subject, also contribute to better development of surgical care for these diseases in the African context.

## Introduction

Non-communicable diseases (NCD), including arterial hypertension (HTN), cardiovascular disease (CVD), diabetes mellitus (DM), and chronic kidney disease (CKD), are diseases with high prevalence and mortality rates that account for 72% of global mortality; 85% of which are from low- and middle-income countries (LMICs)^[1]^. In African countries, for example, they have placed a significant burden on healthcare systems due to lifestyle risk factors and insufficient interventions^[2]^.

**HIGHLIGHTS**

- Non-communicable diseases (NCDs) significantly burden medical services globally, especially in African countries, affecting both healthcare providers and regulatory bodies in surgical specialties.
- Access to surgical services for NCDs in Africa is hindered by several factors, including financial constraints, inadequate infrastructure, and insufficient surgical training.
- Suggested measures to enhance surgical care for NCDs in Africa include international collaborations, improved education and training for professionals, and the use of mobile technology tools.
- The discussion emphasizes the need for multi-professional actions, prevention policies, governmental support, and ongoing research to improve access to surgical care for NCDs in Africa.

In sub-Saharan Africa (SSA), the burden of NCDs increased drastically between 1990 and 2017 reaching 67%, rising from about 90.6 million to 151.3 million, thus putting additional pressure on healthcare services^[3]^. However, the population growth in SSA that increased from about 465.72 million in 1990 to about 977.49 million in 2017 according to Y charts was important factor when considering the 67% increase, but was not the only contributing factor .Urbanizations, lifestyle changes and improved healthcare that lead to longer life span were factors that had a role in this rise as well^[3,4]^. For this reason, concerted action must be taken, along with political will, to prevent, delay the onset of, and manage NCDs effectively^[5]^. NCDs can be managed through surgical approaches, particularly in high-income countries^[6]^. However, due to challenges in understanding NCDs, a multisystemic approach, such as the application of systems medicine, is warranted^[7,8]^. Despite the significance of NCDs in Africa and their impact on the healthcare system, there is still a gap in research addressing surgical care for NCDs. First, better epidemiological data, along with research focusing on screening and disease prevention, is essential^[9]^. Second, existing research does not address the effectiveness and cost of care provided to individuals suffering from NCDs^[10]^. Moreover, further studies are needed to develop and utilize policies related to NCDs across different African nations^[11]^. Developing countries are facing big challenges in dealing with both NCDs and already existing communicable diseases^[12,13]^. Because NCDs account for 75% of this burden, this is considered a heavy strain on the healthcare systems of LMICs^[13]^. They affect many aspects besides direct medical costs, such as productivity, poverty and financial strain^[13]^, with lower-income individuals paying very high health expenditure due to NCDs^[14]^. However, it is very hard to differentiate between communicable and non-communicable diseases, and if there is a criterion to do so, it may be very misleading because they share many common risk factors and interconnected origins^[15]^. Hence, due to this complex relationship between NCDs and communicable diseases, healthcare systems, especially primary healthcare must work very hard to address both diseases simultaneously^[12]^.

This study aims to state the challenges that prevent providing appropriate surgical management of NCDs in African countries, and to set preventive methods and suggest surgical approaches focused on NCD treatment to improve healthcare in African countries.

## Non-communicable diseases: the African context

The burden of NCDs, including CKD, cancer, DM, and CVD, is steadily rising in Africa, particularly within SSA. There is also a corresponding rise in the need for surgical intervention, placing stress on the already strained local healthcare systems^[16]^. This significantly contributes to the high morbidity and mortality experienced in this region compared to developed nations^[2]^. A projection study on the global burden of disease reported that mortality caused by NCDs will increase by 10% between 2002 and 2030^[17]^.This was further elucidated by a large scoping review on NCDs in SSA, showing that NCDs will surpass infectious diseases rates by 2030^[18]^. The World Health Organization (WHO) progress monitor of NCDs estimated that between 50% and 88% of mortalities are attributed to NCDs in seven small African countries. In 2022, the WHO regional director for Africa stated that NCDs contribute to more than one-third of deaths in Africa, with most occurring in younger people (i.e., under 70 years) based on available evidence^[19]^.

CVDs account for a substantial proportion of NCDs in LMICs^[20,21]^. In Africa, CVDs account for 38.3% of NCD deaths, which is the largest contributor^[22]^. Despite reductions in CVD morbidity and mortality in developed nations, there has been an increase in this trend in Africa due to poor preventive measures and late detection of these diseases. As a result, these regions require advanced care and surgical interventions, which are limited in Africa^[21]^. Over 6 billion people do not have access to cardiac surgery, with LMICs accounting for a larger percentage of these individuals due to limited infrastructure and personnel. For example, in Ethiopia, there are only five cardiac surgeons and four centers that carry out cardiac surgeries for a population of more than 120 million, with the waiting list for cardiac surgery estimated at 150 000 people^[23]^. The growing demand for surgical services due to NCDs has a ripple effect on Africa’s already challenged healthcare system, resulting in several critical disadvantages. These include:

- Increased demand for the limited availability of surgical services: Advanced NCDs in Africa often require surgical intervention. The steady rise of NCDs in Africa is typically accompanied by an increased demand for surgical services. However, due to the limited availability of such services, there is a delay in the management of patients needing advanced care, which exacerbates NCD-related mortality and morbidity and widens disparities in healthcare access^[21,24]^.
- Strain on healthcare infrastructure: Surgical facilities, equipment, and human resources are under significant strain. NCDs require comprehensive continuous care over an extended period. In advanced cases where more trained medical professionals and technologies are necessary, the added stress on an already fragile healthcare system leads to suboptimal patient management and poorer health outcomes. A study that explored the resources needed to provide optimal cardiac surgery in Namibia, Zambia, and Uganda showed that these African countries have adequate facilities and surgical expertise; however, they lack vital support staff and essential materials. Unlike countries lacking all resources, these three African countries have an estimated waiting list of 60–500 people^[25]^.
- Increased economic burden: A recent study by Harvard University and the World Economic Forum projected that over the next two decades, the rise of NCDs and the advancement of management services, such as complex surgical interventions, will impose a financial burden of 47 trillion USD on the global economy. This is approximately 75% of the global total gross domestic product^[24]^. As a result, LMICs will experience significant shortfalls in healthcare budgets, leading to limited healthcare funding, inadequate infrastructure, and rising costs, ultimately resulting in substandard surgical services and poor patient outcomes.
- The rapid increase in unmet surgical needs and the relatively limited access to surgical care in Africa are due to several barriers. These challenges can be classified into the 4 “As”: accessibility, affordability, availability, and acceptability.
- Accessibility: Several factors hamper access to surgical care, including limited health education, restricted public health awareness of available treatments, long waiting times, and poor communication with affected populations, discouraging people from seeking medical attention. Additionally, a disjointed healthcare system and inadequate transportation options limit healthcare accessibility^[26-28]^.
- Affordability: The relatively high cost of surgical procedures compared to the minimum wage of the population makes surgical services unaffordable. For instance, amputation secondary to DM accounts for 36.9% of the total amputation in SSA^[29]^. The cost of amputation secondary to DM in a public hospital in Nigeria was estimated to be 175 USD and this was approximately one-fifth of the total amount in managing any stage of DM requiring amputation^[30]^. This suggests that surgical services required in managing NCDs elevate the costs, thereby complicating the financial landscape of NCDs. The minimum wage in Nigeria is 30 000 naira, approximately 19 USD, although it was recently increased to 70 000 naira (43 USD), but this has not been implemented in most sectors^[31]^. The wide contrast between the minimum wage and the cost of surgical services highlights the significant disparities in healthcare affordability and access. Poor remuneration of healthcare workers and expensive surgical equipment further impede access to surgical care in LMICs^[27,32]^.
- Availability: The shortage of skilled workers and inadequate surgical facilities contribute to poor surgical practices in LMICs^[27]^.
- Acceptability: In Africa, a significant portion of the population attributes diseases to spiritual causes due to traditional beliefs, which prevents people from seeking appropriate intervention in healthcare facilities. Furthermore, stigma stemming from fear and cultural perception of invasive therapy hinders the use of surgical services. Finally, poor patient outcomes and misconceptions regarding surgical efficacy further discourage individuals from seeking care^[27,28]^.

These multifactorial barriers collectively impede proper surgical interventions, perpetuating disparities in access to essential surgical care for NCDs across Africa.

## Surgical interventions and NCD management in Africa

The accessibility of surgical departments across the African continent plays a pivotal role in the surgical management of NCDs. One study revealed that major hospitals were located two hours away from most patients in SSA^[33]^. Another study highlighted that some hospitals, such as those in Ethiopia, exhibited significant variations in infrastructure, accessibility and resources^[34]^. Geographic accessibility remains a major barrier, restricting both patients from reaching hospitals and surgical resources from reaching healthcare professionals, thereby negatively impacting NCD management outcomes^[35]^. Furthermore, a study in Ghana illustrated that a critical issue hampering NCD management was the lack of adequately trained surgeons in tertiary care centers^[36]^. As previously mentioned, the burden that NCDs place on healthcare systems is rising in Africa, especially when considering the shortage of surgeons in the region^[37,38]^. Therefore, efficient solutions must be implemented, such as the collaborative academic model of local surgical training in Malawi^[38]^. Moreover, multiple studies have shown that the surgical treatment of certain NCDs is associated with low morbidity rates. It is falsely assumed that non-hospital settings have higher rates of infection; however, studies clarify that lower infection rates are possible when surgeries are conducted by skilled and experienced doctors following appropriate infection control measures, even in resource-limited conditions. Therefore, higher infection rates are not necessarily associated with non-hospital environments^[39,40]^.Such clinical environments include physician’s clinics, office-based laboratories, ambulatory surgical centers, and patients’ homes. The need for major post-traumatic vascular surgery in emergency scenarios is increasing^[37]^. Cases of intussusception are frequently observed, especially in younger populations, and the increasing surgery rates for such conditions must be accounted for when discussing NCDs^[41]^. Additionally, both acute and chronic surgical abdominal conditions, such as colorectal cancer resection, are on the rise in Africa^[41]^. Appendicitis, another common condition, can be resolved through laparoscopic or open appendectomy^[42]^. A recent study reported that the most common surgical procedures performed in SSA include caesarean sections, laparotomies, and hernia repairs, along with less frequent surgeries involving orthopedics, plastic surgery, and neurosurgery^[43]^. The same study noted the number of procedures performed at district hospitals offering both elective and emergency surgeries in 2021 ranged from 239 to 5233^[43]^. Despite these advancements, significant challenges remain in providing surgical care for NCDs in Africa, particularly concerning a lack of infrastructure and resources^[44]^. For instance, in Nigeria, the high prevalence of HTN and DM has prompted medical initiatives aimed at improving access to care for all patients, regardless of disparities^[45]^. However, the high rates of postoperative complications, morbidity, and mortality indicate that these efforts have been largely unsuccessful^[46]^. All prior research underscores the obstacles and challenges related to the surgical management of NCDs and points to a singular goal: enhancing infrastructure, resources, surveillance systems, and accessibility to achieve effective surgical care management for NCDs in Africa.

## Obstacles to successful surgical care for NCDs in Africa

In SSA, access to surgical care in various settings – beyond just NCDs – is limited. Countries in SSA, such as Ghana, Kenya, Rwanda, Tanzania, and Uganda, face shortages of healthcare workers, insufficient training, and gaps in hospital infrastructure^[46]^. These barriers to surgical care are encompassed in “The Three Delays Framework,” which identifies three stages that delay access to surgical care: difficulties in seeking care, challenges in reaching healthcare facilities, and delays in receiving quality health services. A study in Ghana revealed that inadequate care was the main barrier for patients with surgical conditions^[29]^. In Uganda, a study found that 65% of patients who reached the Mulago National Referral Hospital eye clinic with cataracts (an NCD that requires surgical treatment) experienced surgery delays due to financial constraints or their perception that surgery was not necessary^[47]^.

**Figure 1.**
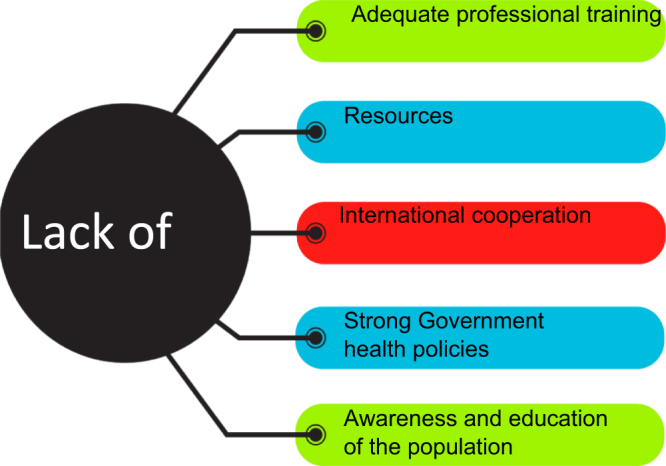
Main barriers to successful surgical care for NCD in SSA.

According to the WHO, NCDs encompass five major categories: CVD, cancer, DM, mental health disorders, and chronic respiratory diseases^[48]^. As cancer becomes increasingly prevalent due to multifactorial environmental and clinical etiologies, it is viewed as a catastrophe across SSA. Although obstacles persist in achieving optimal outcomes, such as inadequate resource management and a lack of experienced healthcare professionals, surgical techniques remain among the most cost-effective therapeutic procedures for solid tumors. Enhancing medical education and training – supported by high-income nations – and developing more robust health policies with government support are essential for creating a safe and affordable surgical oncology system in SSA^[49]^ (Fig. 1).

Chronic respiratory diseases, particularly chronic obstructive pulmonary disease (COPD), have significantly increased over the last 20 years in SSA, with morbidity and mortality rates rising due to post-surgical complications^[50]^. Although surgical intervention is uncommon for COPD, it is considered in certain advanced stages^[51]^.

Amputation is a common surgical procedure for populations affect by DM, and remains a significant complication of the disease globally, including in SSA^[29]^. To address this issue, patients must be properly educated about foot care, made aware of the potential consequences of poor glycemic control, and encouraged to regularly examine their feet. This proactive approach is essential in preventing lower limb amputations, which contribute to higher mortality and morbidity rates^[52]^.

## Improving Surgical Services for NCDs in Africa

A multifaceted approach is required to enhance surgical services for NCDs in Africa, primarily focusing on addressing the challenges that hinder quality surgical care.

- Improving the financial and infrastructure needs of surgical care: Several strategies can be employed to increase funding for surgical services in this region. Leveraging public-private partnerships and collaborating with international organizations may improve infrastructure development and equipment maintenance, thereby bridging the gap between finances and sustainability^[6]^. Health insurance also plays a crucial role in reducing disparities in access to surgical care in LMICs by developing inclusive policies, streamlining financing methodologies, improving strategic purchasing, and ensuring coverage for marginalized populations^[32,53]^.
- Improving the standard of skilled workers: Human resources are fundamental to adequate surgical care, especially in SSA, where surgical personnel are limited. Proper training and fair compensation, combined with personal and financial incentives, can aid in retaining qualified healthcare providers and prevent the exodus of professionals due to “brain drain”^[54]^. Developing structured training programs with international recognition and offering financial rewards are essential strategies to ensure that skilled professionals continue delivering quality care in Africa. International collaborations with developing countries can also help elevate the standard of surgical techniques and training^[55]^.
- Digital health in surgical care for NCDs: Telesurgery has proven effective in improving surgical care in Africa by reducing the need for long-distance travel while enhancing teamwork among surgeons. This synergy helps optimize the output of the limited surgeons available and decreases the risk of infections, particularly in regions prone to infectious disease endemics^[56-58]^. A multidisciplinary approach is needed to integrate telesurgery into the African healthcare system. Aligning national health strategies with WHO guidelines, with a focus on surgical care, will help advance digital health in African surgical services. However, a systematic approach is necessary to improve surgical infrastructure before telesurgery can be widely implemented^[58]^.
- Collaboration with high-income countries: Collaborating with developed countries is crucial to improve surgical care for NCDs in Africa. High-income countries possess advanced technologies and expertise that can significantly improve patient outcomes. Through partnerships, knowledge exchange, and resource sharing, African nations can better address the growing burden of NCDs^[59]^. Developed countries can also provide quality training programs and continuous education for African surgeons, thereby enhancing their skills. Additionally, joint research initiatives can lead to the innovative development of surgical techniques and therapies tailored to African populations^[55,59-61]^.
- Role of Indigenous African Surgical Colleges: These colleges are very important in improving the capacity of surgery across the continent, by providing training programs specific for each region. They also help standardize surgical practices, ensuring that surgeons follow the current guidelines in their practices. In addition to this, they help in organizing conferences, fostering research, and collaboration with high-income countries, to improve healthcare across Africa^[62]^. For instance, the West Africa College of Surgeons is a leading indigenous college in Africa, with more than 8000 surgical specialists and several trainees across different specialties covering over 18 countries. This highlights the importance of this college and the potential for great impact if scaled up^[63]^. West Africa College of Surgeons is fast becoming the role model for upcoming indigenous surgical colleges across Africa^[31]^. Expanding indigenous Africa Colleges will require a rigorous multifaceted approach, comprising innovation, collaboration, and funding. Reinforcing these colleges will help in building a self-sufficient surgical workforce, required for addressing the burden of surgical care.

## Preventing and fighting NCDs in Africa

Across the world, surgery addresses approximately one-third of NCD cases^[64]^. Surgical care plays a critical role in NCD management, though it has often been overlooked in recent NCD guidelines. Major examples demonstrating its necessity include cataract surgery for treating blindness and cancer excision as a surgical approach^[65]^. In Africa, it is no different; cataracts, which are classified as an NCD and can be treated surgically, remain a leading cause of blindness on the continent^[66]^. NCDs have become a significant burden over the past 20 years, especially in SSA^[67]^.

In this context, policy gaps in NCD management result in inadequate support and care, limited medication affordability, and the social and economic obstacles associated with sedentary lifestyles, including drug and alcohol misuse and unhealthy eating habits^[68]^. Additionally, there is a lack of multi-stakeholder involvement across sectors like agriculture, education, security, and other areas beyond healthcare^[68]^.

There is also a noted scarcity in the effective implementation of existing policies, leading to suboptimal outcomes. This gap is often due to insufficient government funding and a lack of recognition of the importance of NCDs as a public health priority. Then it results in a lack of political and financial support. To address this, countries should be encouraged to develop stronger prevention policies for NCDs^[69]^.

The implementation of effective interventions must be a top priority across all nations. Achieving optimal postoperative outcomes requires adequate support from governments and industries that influence NCD risk factors, such as the food, alcohol, and tobacco sectors. It is also critical to require through policies that these industries engage to address the adverse health effects caused by their products^[69]^.

The WHO’s global NCD action plan has provided valuable guidance for countries in developing prevention policies. However, difficulties in the policy implementation persist due to limited resources, political commitment, and technical capacity^[69]^. An illustration of this issue was presented in a study published in 2018 that evaluated NCD prevention policies in five African countries: Kenya, South Africa, Cameroon, Nigeria, and Malawi^[70]^. In Kenya, policies were established to address the main risk factors for NCDs, such as sedentary lifestyles, inadequate diet, and alcohol and tobacco consumption, alongside action plans and specific legislation to manage these factors. In South Africa, policies have been implemented to regulate alcohol advertising and tobacco consumption, but obstacles to policy creation have arisen due to a lack of agreement between different sectors, particularly between trade and health sectors^[70-72]^.

Cameroon, despite confirming its adherence to the World Health Organization Framework Convention on Tobacco Control (FCTC) and establishing policies to reduce tobacco use, still faces challenges that complicate the efficiency and full implementation of these policies, as the government maintains subsidy measures for tobacco farmers. In Nigeria, policies aimed at promoting health and controlling tobacco consumption have been created, with recognition of the importance of a multi-sectoral approach to NCD prevention. However, obstacles such as a lack of commitment from political leaders and difficulties in resource allocation persist^[70,73]^.

Finally, in Malawi, various sectors and stakeholders have been involved in creating policies, especially those promoting physical activity and controlling tobacco use, but resource allocation and policy implementation remain inadequate. Overall, it was observed that while the development of NCD prevention policies in these African countries represents progress, barriers such as insufficient resources, a lack of political leadership committed to the issue, and limited collaboration across multiple sectors still need to be addressed for effective policy implementation^[70,73]^.

Various strategies can be applied to achieve the effective implementation of NCD policies. Examples include establishing organized mechanisms that promote multi-sectoral collaboration and developing well-defined guidelines for multi-sectoral action. Additionally, training professionals across different sectors of society, ensuring adequate funding for policy establishment, encouraging the commitment of political leaders to the cause, and raising public awareness of the issue are important strategies^[70,73]^.

Moreover, creating models for evaluating and monitoring the implementation of these policies is essential^[70]^. In addition, mobile health technologies can help strengthen healthcare systems in fighting NCDs in SSA and in other African countries as well^[74]^.

## Future perspectives

Studies suggest that surgical procedures can assist not only in the management but also in the diagnosis and palliation of patients with NCDs. However, surgical teams in disaster settings are often not prepared to handle a large number of patients with NCDs^[75]^.

To overcome the current challenge of NCDs, a multifaceted approach is required. This includes humanitarian efforts focused on providing appropriate training for healthcare teams, implementing strategies that emphasize prevention, ensuring continuity of care, promoting lifestyle modifications, considering the costs of treatment, and minimizing interruptions in therapy^[75,76]^.

The use of medical digital technology applications for NCDs is increasing^[77]^. Health technologies and medical devices can play a key role in the prevention and control of NCDs. However, for these technologies to be effective, they must meet certain criteria, such as affordability and ease of implementation. It is also crucial that healthcare professionals receive structured education and training in their use^[78]^. Although, digital solutions may not be as effective for elderly populations with chronic diseases, as they tend to be less engaged with digital platforms^[76,77,79]^.

Promoting health is an essential component of NCD management. This can be achieved by encouraging healthy lifestyles that target associated risk factors, such as promoting good nutrition and smoking cessation. Modern strategies for managing NCDs should involve community and governmental engagement at both national and international levels to promote awareness of human and environmental health. Innovations and research can also contribute on many different levels for the development of a healthier world^[80,81]^.

According to the WHO, there are numerous needs related to NCDs, but resources are scarce. This raises the reflection that while humanitarian efforts are important, they are a temporary fix, not a long-term solution. Humanitarian aid is crucial, especially in providing appropriate training, but there are additional obstacles to address, such as the lack of local resources, limited patient resources, and complex government support situations^[82]^.

## Conclusion

The rising prevalence of NCDs poses an increasing threat to Africa’s healthcare systems. However, addressing all challenges, such as financial constraints, policy gaps, inadequate infrastructure, limited accessibility, and insufficient training for surgeons, can improve surgical management of NCDs.

Although the majority of treatments for NCDs are non-surgical, surgical treatment plays a key role in managing many NCDs and should be included in new NCD management guidelines to emphasize the need for improved access to and quality of surgical care, especially in underserved areas such as SSA. Furthermore, ongoing and continuous research is crucial to provide the best possible surgical care for NCDs.

## Data Availability

Data availability is not applicable to this article as no new data were created or analyzed in this study.

## References

[1] Mapesi H Paris DH. Non-communicable diseases on the rise in sub-Saharan Africa, the underappreciated threat of a dual disease burden. Praxis (Bern 1994) 2019;108:997–1005. 31771492 10.1024/1661-8157/a003354

[2] Juma K Juma PA Shumba C. Non-communicable diseases and urbanization in African cities: a narrative review. Public Health in Developing Countries – Challenges and Opportunities. IntechOpen; 2020.

[3] Gouda H Charlson F Sorsdahl K. Burden of non-communicable diseases in sub-Saharan Africa, 1990-2017: results from the global burden of disease study 2017. Lancet Global Health 2019;7:e1375–e1387. 31537368 10.1016/S2214-109X(19)30374-2

[4] Phillips DR. Urbanization and human health. Parasitology 1993;106:S93–107. 8488075 10.1017/s0031182000086145

[5] Nojilana B Bradshaw D Pillay-van Wyk V. Persistent burden from non-communicable diseases in South Africa needs strong action. S Afr Med J 2016;106:436–37. 10.7196/SAMJ.2016.v106i5.10776 27138654

[6] Jarnheimer A Kantor G Bickler S. Frequency of surgery and hospital admissions for communicable diseases in a high-and middle-income setting. Br J Surg 2015;102:1142–49. 26059635 10.1002/bjs.9845

[7] Uwishema O Boon P. Bridging the gaps: addressing inequities in neurological care for underserved populations. Eur J Neurol 2025;32:e70073. 39912252 10.1111/ene.70073 PMC11799841

[8] Bousquet J Anto JM Sterk PJ. Systems medicine and integrated care to combat chronic noncommunicable diseases. Genome Med 2011;3:1–2. 21745417 10.1186/gm259 PMC3221551

[9] Maher D Sekajugo J Harries AD. Research needs for an improved primary care response to chronic non-communicable diseases in Africa. Trop Med Int Health 2010;15:176–81. 20002618 10.1111/j.1365-3156.2009.02438.x

[10] Vorkoper S Kupfer LE Anand N. Building on the HIV chronic care platform to address noncommunicable diseases in sub-Saharan Africa: a research agenda. Aids 2018;32:S107–13. 29952796 10.1097/QAD.0000000000001898 PMC6438180

[11] Kassa MD Grace JM. Noncommunicable diseases prevention policies and their implementation in Africa: a systematic review. Public Health Rev 2022;42:1604310. 35295954 10.3389/phrs.2021.1604310 PMC8865333

[12] Boutayeb A Boutayeb S. The burden of non communicable diseases in developing countries. Int J Equity Health 2005;14:4–2. 10.1186/1475-9276-4-2 PMC546417 15651987

[13] Nikolic IA. Chronic emergency: Why NCDS matter [Internet]. Health, Nutrition and Population; 2011 [cited 2024 Mar 13]. https://documents.worldbank.org/en/publication/documentsreports/documentdetail/267551468148765055/chronic-emergencywhy-ncds-matter

[14] Haakenstad A Coates MM Buhkman G. Comparative health systems analysis of differences in the catastrophic health expenditure associated with non-communicable vs communicable diseases among adults in six countries. Health Policy Plan 2022;37:1107–15. 35819006 10.1093/heapol/czac053 PMC9557357

[15] Mohan P Mohan SB Dutta M. Communicable or noncommunicable diseases? Building strong primary health care systems to address double burden of disease in India. J Family Med Prim Care 2019;8:326–29. 30984632 10.4103/jfmpc.jfmpc_67_19 PMC6436242

[16] Dalal S Beunza JJ Volmink J. Non-communicable diseases in sub-Saharan Africa: what we know now. Int J Epidemiol 2011 Aug 1;40:885–901. 21527446 10.1093/ije/dyr050

[17] Mathers CD Loncar D Samet J. Projections of global mortality and burden of disease from 2002 to 2030. PLoS Med 2006;3:e442. 17132052 10.1371/journal.pmed.0030442 PMC1664601

[18] Mudie K Jin MM Tan Kendall L. Non-communicable diseases in sub-Saharan Africa: a scoping review of large cohort studies. J Glob Health 2019;9:020409. 31448113 10.7189/jogh.09.020409 PMC6684871

[19] WHO Regional Office for Africa [Internet]. 2024 [cited 2024 Mar 24]. Deaths from noncommunicable diseases on the rise in Africa. Available from: https://www.afro.who.int/news/deaths-noncommunicable-diseases-rise-africa

[20] Noncommunicable diseases. King’s Global Health Institute | King’s College London [Internet]. [cited 2024 Mar 26]. Available from: https://www.kcl.ac.uk/kghi/research/noncommunicable-diseases

[21] Byiringiro S Nyirimanzi N Mucumbitsi J. Cardiac surgery: increasing access in low- and middle-income countries. Curr Cardiol Rep 2020;22:37. 32430786 10.1007/s11886-020-01290-5

[22] Minja NW Nakagaayi D Aliku T. Cardiovascular diseases in Africa in the twenty-first century: gaps and priorities going forward. Front Cardiovasc Med 2022;9:1008335. 36440012 10.3389/fcvm.2022.1008335 PMC9686438

[23] Argaw S Genetu A Vervoort D. The state of cardiac surgery in Ethiopia. JTCVS Open 2023;14:261–69. 37425461 10.1016/j.xjon.2023.03.001 PMC10328795

[24] Temu F Leonhardt M Carter J. Integration of non-communicable diseases in health care: tackling the double burden of disease in African settings. Pan Afr Med J 2014;18:202. 25419329 10.11604/pamj.2014.18.202.4086 PMC4237574

[25] Forcillo J Watkins DA Brooks A. Making cardiac surgery feasible in African countries: experience from Namibia, Uganda, and Zambia. J Thorac Cardiovasc Surg 2019;158:1384–93. 30819574 10.1016/j.jtcvs.2019.01.054

[26] Mac Quene T Smith L Odland ML. Prioritising and mapping barriers to achieve equitable surgical care in South Africa: a multi-disciplinary stakeholder workshop. Glob Health Action 2022;15:2067395. 35730572 10.1080/16549716.2022.2067395 PMC9225684

[27] Ologunde R Maruthappu M Shanmugarajah K. Surgical care in low and middle-income countries: burden and barriers. Int J Surg 2014;12:858–63. 25019229 10.1016/j.ijsu.2014.07.009

[28] Grimes CE Bowman KG Dodgion CM. Systematic review of barriers to surgical care in low-income and middle-income countries. World J Surg 2011;35:941–50. 21360305 10.1007/s00268-011-1010-1

[29] Boateng D Ayellah BB Adjei DN. Contribution of diabetes to amputations in sub-Sahara Africa: a systematic review and meta-analysis. Prim Care Diabetes 2022;16:341–49. 35305899 10.1016/j.pcd.2022.01.011

[30] Danmusa UM Terhile I Nasir IA. Prevalence and healthcare costs associated with the management of diabetic foot ulcer in patients attending Ahmadu Bello University Teaching Hospital, Nigeria. Int J Health Sci (Qassim) 2016;10:219. 27103904 PMC4825895

[31] Minimum wage in Nigeria: who and who go earn 70,000 naira new minimum wage – BBC News Pidgin [Internet]. [cited 2024 Oct 31]. Available from: https://www.bbc.com/pidgin/articles/cd10pjj944lo

[32] Expanding surgical access in Africa through improved health: medicine [Internet]. [cited 2024 Mar 29]. Available from: https://journals.lww.com/md-journal/fulltext/2024/03150/expanding_surgical_access_in_africa_through.64.aspx 10.1097/MD.0000000000037488 PMC10939550 38489736

[33] Juran S Broer PN Klug SJ. Geospatial mapping of access to timely essential surgery in sub-Saharan Africa. BMJ Glob Health 2018;3:e000875. 10.1136/bmjgh-2018-000875 PMC6104751 30147944

[34] Chao TE Burdic M Ganjawalla K. Survey of surgery and anesthesia infrastructure in Ethiopia. World J Surg 2012;36:2545–53. 22851147 10.1007/s00268-012-1729-3

[35] Dell AJ Kahn D. Geographical maldistribution of surgical resources in South Africa: a review of the number of hospitals, hospital beds and surgical beds. S Afr Med J 2017;107:1099–105. 29262964 10.7196/SAMJ.2017.v107i12.12539

[36] Abdullah F Choo S Hesse AA. Assessment of surgical and obstetrical care at 10 district hospitals in Ghana using on-site interviews. J Surg Res 2011;171:461–66. 20691981 10.1016/j.jss.2010.04.016

[37] Archampong EQ. Surgery in developing nations. Br J Surg 2006;93:516–17. 16607681 10.1002/bjs.5317

[38] Qureshi JS Young S Muyco AP. Addressing Malawi’s surgical workforce crisis: a sustainable paradigm for training and collaboration in Africa. Surgery 2013;153:272–81. 23063312 10.1016/j.surg.2012.08.004

[39] Ameh EA Abantanga FA Birabwa-Male D. Surgical aspects of bacterial infection in African children. Semin Pediatr Surg 2012;21:116–24. 22475117 10.1053/j.sempedsurg.2012.01.004

[40] Gil J Rodríguez JM Gil E. Surgical treatment of endemic goiter in a nonhospital setting without general anesthesia in Africa. World J Surg 2014;38:2212–16. 24728536 10.1007/s00268-014-2553-8

[41] Archampong EQ Naaeder SB Darko R. Changing pattern of intestinal obstruction in Accra, Ghana. Hepato-gastroenterology 2000;47:185–93. 10690607

[42] Clegg-Lamptey J Naaeder S. Appendicitis in Accra: a contemporary appraisal. Ghana Med J 2003;37:52–56.

[43] Bentounsi Z Sheik-Ali S Drury G. Surgical care in district hospitals in sub-Saharan Africa: a scoping review. BMJ Open 2021;11:e042862. 10.1136/bmjopen-2020-042862 PMC7996654 33766839

[44] Dare AJ Onajin-Obembe B Makasa EM. A snapshot of surgical outcomes and needs in Africa. Lancet 2018;391:1553–54. 29306588 10.1016/S0140-6736(18)30002-3

[45] Oruche U Liu J Otey T. Engaging communities to improve healthcare for non-communicable diseases. ENGAGE! Co-created Knowledge Serving the City 2020;1.

[46] Friedrich MJ. NTDs added to African leaders malaria alliance scorecard. JAMA 2018;319:1085. 10.1001/jama.2018.2365 29558560

[47] Sebabi FO Okello WO Nakubulwa F. Factors associated with delayed uptake of cataract surgery among adult patients at Mulago National Referral Hospital, Uganda. Afr Health Sci 2021;21:1259–65. 35222590 10.4314/ahs.v21i3.36 PMC8843270

[48] Noncommunicable Diseases [Internet]. WHO | Regional Office for Africa. 2024 [cited 2024 Mar 28]. Available from: https://www.afro.who.int/health-topics/noncommunicable-diseases#:~:text=These%20five%20main%20NCDs%20are

[49] Taliente F Kisekka PK Ssembuusi J. Enhancing surgical oncology in sub-Saharan Africa through international cooperation. Eur J Surg Oncol 2023;49:918–20. 36690532 10.1016/j.ejso.2023.01.015

[50] Abdool-Gaffar MS Calligaro G Wong ML. Management of chronic obstructive pulmonary disease-A position statement of the South African Thoracic Society: 2019 update. J Thorac Dis 2019;11:4408–27. 31903229 10.21037/jtd.2019.10.65 PMC6940223

[51] Information NC for B, Pike USNL of M 8600 R, MD B, USA 20894. Chronic obstructive pulmonary disease (COPD): surgical procedures for the treatment of COPD [Internet]. Institute for Quality and Efficiency in Health Care (IQWiG); 2019. Available from: https://www.ncbi.nlm.nih.gov/books/NBK543216/

[52] Owolabi EO Chu KM. Knowledge, attitude and perception towards lower limb amputation amongst persons living with diabetes in rural South Africa: a qualitative study. Afr J Prim Health Care Fam Med 2022;14:e1–e10. 10.4102/phcfm.v14i1.3398 PMC9623825 36226936

[53] Mugisha N Uwishema O Noureddine R. Utilization of mobile surgical units to address surgical needs in remote African communities: a narrative review. BMC Surg 2024;24:304. 39395989 10.1186/s12893-024-02596-9 PMC11470661

[54] Akenroye OO Adebona OT Akenroye AT. Surgical care in the developing world-strategies and framework for improvement. J Public Health Africa 2013;4:e20. 10.4081/jphia.2013.e20 PMC5345438 28299109

[55] Du Plessis PJ Leventer M Krekels G. Outcomes of Mohs micrographic surgery at the American society for dermatologic surgery international traveling mentorship program international Mohs fellowship recognition units: a retrospective survey of 5889 cases from South Africa, Romania, and the Netherlands. Dermatol Surg 2019;45:S155–S162. 31764300 10.1097/DSS.0000000000002251

[56] Digital health systems to improve perioperative patient outcomes – phase 1 – APPRISE [Internet]. [cited 2024 Mar 29]. Available from: https://globalperioperativecriticalcare.org/digital-health-systems-to-improve-perioperative-patient-outcomes-phase-1/

[57] Choi PJ Oskouian RJ Tubbs RS Telesurgery: past, present, and future. Cureus [Internet]. 2018 May 31 [cited 2024 Mar 29]; Available from: https://www.cureus.com/articles/12751-telesurgery-past-present-and-future 10.7759/cureus.2716 PMC6067812 30079282

[58] Mehta A Andrew Awuah W Tunde Aborode A. Telesurgery’s potential role in improving surgical access in Africa. Ann Med Surg 2022;82:104511. 10.1016/j.amsu.2022.104511 PMC9577435 36268331

[59] Kushner AL Kamara TB Groen RS. Improving access to surgery in a developing country: experience from a surgical collaboration in Sierra Leone. J Surg Educ 2010;67:270–73. 20816367 10.1016/j.jsurg.2010.05.004

[60] Breedt DS Odland ML Bakanisi B. Identifying knowledge needed to improve surgical care in Southern Africa using a theory of change approach. BMJ Glob Health 2021;6. Available from https://www.ncbi.nlm.nih.gov/pmc/articles/PMC8208008/. 10.1136/bmjgh-2021-005629 PMC8208008 34130990

[61] Inshutiyimana S Uwishema O Ramadan N. Challenges and opportunities for Mohs surgery implementation in African healthcare systems. BMC Surg 2024;24:287. 39367373 10.1186/s12893-024-02588-9 PMC11451233

[62] Omigbodun A. The membership certification of the West African College of Surgeons and its relevance to the needs of the West African sub-region. J West Afr Coll Surg 2012;2:83–87. 25452996 PMC4240233

[63] Sule AZ Alayande BT Ojo EO. The history and evolution of the West African College of Surgeons/Jos University Teaching Hospital Trauma Management Course. World J Surg 2023;47:1919–29 37069318 10.1007/s00268-023-07004-6 PMC10109223

[64] Mikkelsen B Casolino R. The pathway for surgery as an integral part of attaining universal health coverage. Lancet Oncol 2023;24:1298–301. 37924821 10.1016/S1470-2045(23)00566-1

[65] Zadey RS and S. (2023) New guidelines on non-communicable diseases are welcome, but where is surgical care? The Wire Science. Available at: https://science.thewire.in/health/new-guidelines-non-communicable-diseases-surgical-care/ (Accessed: 01 November 2024).

[66] Aboobaker S Courtright P. Barriers to cataract surgery in Africa: a systematic review. Middle East Afr J Ophthalmol 2016;23:145–49. 26957856 10.4103/0974-9233.164615 PMC4759895

[67] Bigna JJ Noubiap JJ. The rising burden of non-communicable diseases in sub-Saharan Africa. Lancet Glob Health 2019;7:e1295–e1296. 31537347 10.1016/S2214-109X(19)30370-5

[68] Amuyunzu-Nyamongo M. Noncommunicable diseases, injuries, and mental health: the triple burden in Africa. Pan Afr Med J 2022;43:167. 36825124 10.11604/pamj.2022.43.167.38392 PMC9941609

[69] Juma PA Wisdom J. Introduction: non-communicable disease prevention policies in six African countries. BMC Public Health 2018;18:955. 30168396 10.1186/s12889-018-5824-8 PMC6117631

[70] Juma PA Mapa-Tassou C Mohamed SF. Multi-sectoral action in non-communicable disease prevention policy development in five African countries. BMC Public Health 2018;18:953. 30168391 10.1186/s12889-018-5826-6 PMC6117629

[71] Mugisha N Uwishema O Noureddine R. Access to specialist plastic surgery in rural vs. urban areas of Africa. BMC Surg 2024;24:418. 39725960 10.1186/s12893-024-02735-2 PMC11670430

[72] Hamitoglu AE Fawaz V Elawad SOM. Trends and outcomes of laparoscopic surgery in low-resource settings: lessons from two African healthcare systems – a narrative review. Health Sci Rep 2024;7:e70304. 39720243 10.1002/hsr2.70304 PMC11667220

[73] Kwan Su Huey A Sengar AS Kazan Z. The role of telemedicine in enhancing surgical care delivery in Africa: a literature review. Health Sci Rep 2024;7:e70264. 39698528 10.1002/hsr2.70264 PMC11653025

[74] Olowoyo P Popoola F Yaria J. Strategies for reducing non-communicable diseases in Africa. Pharmacol Res 2021;170:105736. 34147659 10.1016/j.phrs.2021.105736 PMC8800856

[75] Leff R Selvam A Bernstein R. A review of interventions for noncommunicable diseases in humanitarian emergencies in low- and middle-income countries. Am J Disaster Med 2021;16:297–311. 10.5055/ajdm.2021.0412 35325464

[76] Mugisha N Ghanem L Komi OAI. The screening and management of skin diseases in remote African regions: a narrative review. Postgrad Med J 2024:qgae133. doi:10.1093/postmj/qgae133 39533863

[77] Hussein ESE Al-Shenqiti AM Ramadan RME. Applications of medical digital technologies for noncommunicable diseases for follow-up during the COVID-19 pandemic. Int J Environ Res Public Health 2022;19:12682. 36231982 10.3390/ijerph191912682 PMC9565945

[78] Berumen AV Binello N Thangavelu S. Access to medical technologies for NCD prevention and control. In: Noncommunicable diseases: A compendium. 1st ed. London: Taylor and Francis; 2023: 332-9

[79] Tukur HN Uwishema O Soufan F. The role of NGOs and humanitarian organizations in enhancing surgical capacity in Africa: lessons learned and future directions-a narrative review. Postgrad Med J 2024:qgae137. doi:10.1093/postmj/qgae137 39487799

[80] Budreviciute A Damiati S Sabir DK. Management and prevention strategies for non-communicable diseases (NCDs) and their risk factors. Front Public Health 2020;8:574111. 33324597 10.3389/fpubh.2020.574111 PMC7726193

[81] Uwishema O El Fil S Rupani A. Ethical considerations in surgical research conducted in African LMICs: a comprehensive narrative review. Ann Med Surg (Lond) 2024;86:6568–75. 39525741 10.1097/MS9.0000000000002485 PMC11543183

[82] Inclusion of noncommunicable disease care in response to humanitarian emergencies will help save more lives [Internet]. World Health Organization; [cited 2025 Mar 14]. Available from: https://www.who.int/news/item/27-02-2024-inclusion-of-noncommunicable-disease-care-in-response-to-humanitarian-emergencies-will-help-save-more-lives-1

